# Constituents of Food

**Published:** 1891-06

**Authors:** 


### Constituents of Food.

The amount of solid matter in the different kinds of food used should be kept in mind as completely as possible. The following table exhibits the proportion of solid matter and water in 100 parts each of the following articles of diet :

| Articles. | Solid Matter. | Water. | Articles. | Solid Matter. | Water. |
|---|---|---|---|---|---|
| Wheat | 87 | 13 | Pork | 24 | 76 |
| Peas | 87 | 13 | Codfish | 21 | 79 |
| Rice | 86 | 14 | Blood | 20 | 80 |
| Beans | 86 | 14 | Trout | 19 | 81 |
| Rye | 86 | 14 | Apples | 18 | 82 |
| Corn | 86 | 14 | Pears | 16 | 84 |
| Oat-meal | 74 | 26 | Carrots | 13 | 87 |
| Wheat-bread | 51 | 49 | Beats | 13 | 87 |
| Mutton | 29 | 71 | Milk | 13 | 87 |
| Chicken | 27 | 73 | Oysters | 13 | 87 |
| Lean beef | 26 | 74 | Cabbage | 8 | 92 |
| Eggs | 26 | 74 | Turnips | 7 | 93 |
| Veal | 25 | 75 | Watermellon | 5 | 95 |
| Potatoes | 25 | 75 | Cucumber | 3 | 97 |

